# Long-term oncologic safety of immediate reconstructive surgery in patients with invasive breast cancer: a retrospective matched-cohort study

**DOI:** 10.1186/s12957-021-02450-9

**Published:** 2021-12-20

**Authors:** Yanni Song, Shanshan Sun, Dalin Li, Jiguang Han, Ming Niu, Sai Luo, Haiqian Xu, Rui Huang, Sihang Zhang, Yang Wu, Qiqi Wu, Jing Xiong, Lijun Hao

**Affiliations:** 1 Department of Plastic and Cosmetic Surgery, The First Affiliated Hospital of Harbin Medical University, 141 Yiman Road, Harbin, 150010 China; 2 Department of Breast Surgery, Harbin Medical University Cancer Hospital, 150 Haping Road, Harbin, 150081 China; 3 Department of Pathology, Tianjin Medical University Cancer Hospital, 1 Huanhu Xi Road, Tianjin, 300060 China; 4 Department of General Surgery, The Second Affiliated Hospital of Harbin Medical University, 246 Xuefu Road, Harbin, 150086 China; 5 Department of Epidemiology and Bio-Statistics, School of Public Health, Peking University Health Science Center, 38 Xueyuan Road, Haidian District, Beijing, China; 6 Sino-Russian Medical Research Center, Heilongjiang Academy of Medical Sciences, 157 Baojian Road, Harbin, China; 7 Northern (China) Translational Medicine Research and Cooperation Center, Heilongjiang Academy of Medical Sciences, 157 Baojian Road, Harbin, China

**Keywords:** Breast cancer, Breast reconstruction, Breast conservation surgery, Modified radical mastectomy, Patient satisfaction, Prognosis

## Abstract

**Objective:**

Immediate reconstruction (IR) is a safe and effective surgical treatment for patients with breast cancer. We aimed to assess the prognosis, aesthetic outcomes, and patient satisfaction of IR compared with breast conservation surgery (BCS) and total mastectomy (TM).

**Methods:**

This retrospective matched-cohort study was conducted between May 2005 and December 2014. We established two cohorts according to the tumor (T) size of breast cancer. In the T≤3cm group, cases (IR) and controls (BCS or TM) were matched for age, pathological tumor size, and pathologic nodal status in a 1:1:1 ratio. In the T>3cm group, cases (IR) and controls (TM) were matched with the same factors and ratio. The primary outcome was the 5-year disease-free survival (DFS). The secondary outcome was patient satisfaction and quality of life.

**Results:**

A total of 12,678 breast cancer patients were assessed for eligibility, of which 587 were included (T≤3 cm group: 155 IR vs 155 BCS vs 155 TM; T>3cm group: 61 IR vs 61 TM). In the T≤3 cm cohort, patients who underwent IR had no difference compared with those who underwent BCS or TM regarding the 5-year DFS (*P*=0.539); however, an improved aesthetic satisfaction, psychosocial, and sexual well-being were achieved in the IR group (*P*<0.001). In the T>3 cm cohort, the IR group had a worse median 5-year DFS (*P*=0.044), especially for Her2+ or triple-negative breast carcinoma (TNBC) subtypes compared with the TM group.

**Conclusions:**

IR improves aesthetic satisfaction, psychosocial, and sexual well-being for breast cancer patients with T≤3 cm. For patients with T > 3 cm invasive breast cancer, TM is superior to IR as it predicts a better 5-year DFS.

## Introduction

Breast cancer is the most common malignant disease among women worldwide, with an incidence varies between 27 in 100,000 (Africa and East Asia) and 97 in 100,000 (North America) worldwide [1]. Once diagnosed with early-stage breast cancer, most women would undergo breast conservation surgery (BCS) or total mastectomy (TM) to either partially or completely remove the breast. However, no matter which surgical technique is used, patients will be suffering from breast loss and a decreased quality of life [2].

Immediate reconstructive (IR) surgery, performed simultaneously for patients who have undergone a radical mastectomy, has become increasingly important in recent years. IR is particularly an attractive strategy for breast cancer treatment when BCS is not applicable. IR can benefit patients both physically and psychologically when compared with TM and BCS. For example, IR will improve patients’ self-esteem and overall quality of life, reduce sexual dysfunction, and decrease body image anxieties [3–9]. Survival has always been the primary objective for patients with breast cancer. A previous match-cohort study has identified several associated factors that can predict the prognosis of breast cancer for patients who underwent BCS [10]. However, no studies systematically assessed the long-term oncologic follow-up outcomes of IR applied for patients with different lesion sizes. In addition, whether breast cancer patients with different molecular subtypes can benefit from IR remains unclear. Although some previous studies have reported improved the quality of life and satisfying aesthetic outcomes for patients who underwent either BCS or TM, no matter whether with or without reconstruction [11–15], direct comparisons with respect to survival and safety outcomes between IR, BCS and TM are scarce.

Therefore, we conducted this retrospective matched-cohort study to compare the prognosis, aesthetic outcomes, and complication-related quality of life outcomes of IR, TM, and BCS performed in patients with invasive ductal carcinomas (IDCs) with different tumor sizes. We also examined the association between cancer subtypes and the 5-year DFS of IR in both T≤3 cm and T>3 cm groups.

## Methods

### Study design and patients

**Fig. 1:**
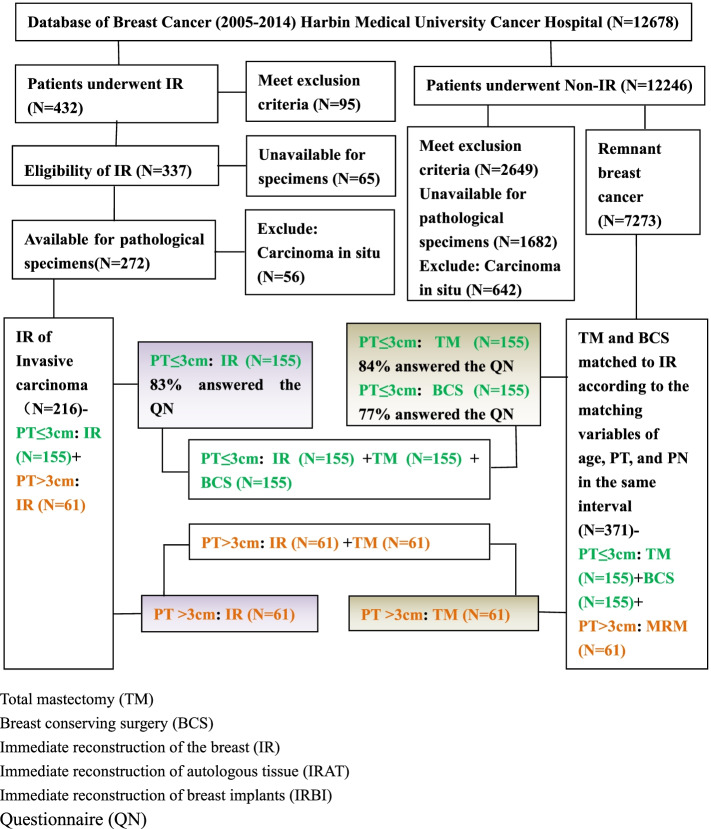
Study object. Patients were selected from the database of the Breast Cancer Center between 2005 and 2014. IR immediate reconstruction, IRBI immediate reconstruction of breast implants, IRAT immediate reconstruction of autologous tissue, BCS breast-conserving surgery, TM total mastectomy

We conducted this retrospective matched cohort study at the Harbin Medical University Cancer Hospital (HMUCH, Heilongjiang, China) between May 2005 and December 2014. The initial entry criteria for cases to be included in this report were the availability of HMUCH data (Fig. 1). Patients were selected from a standardized institutional database if they (1) had histologically or pathologically confirmed invasive breast cancer without distant metastasis or local relapses and (2) had surgical pathological specimens of the primary tumor available for review. Cancer stages were identified according to the American Joint Committee on Cancer [AJCC] TNM staging system [16]. All patients were scheduled with IR. Exclusion criteria included in situ carcinoma, bilateral, or multicentric breast cancer, recurrent cancer, metastatic breast cancer at presentation, a history of invasive breast cancer, other previous tumors, pregnancy, and the death of the patient. Patients who received neoadjuvant chemotherapy and intraoperative electron beam radiotherapy for cancer treatment were also excluded. Patients with T > 3cm lesions were not included either if they undertook neoadjuvant chemotherapy before BCS. This study was approved by the Ethical Committee of Harbin Medical University Cancer Hospital. This study was performed in accordance with the Declaration of Helsinki, and all patients had given the signed informed consent to participate.

### Procedures

We established two different study cohorts according to the tumor (T) size of breast cancer. Cases in the T ≤ 3 cm group were invasive ductal carcinomas patients who underwent IR. Controls were patients who underwent either BCS or TM, matching with cases for age, pathological tumor size (pT), and pathologic nodal status (pN) in a 1:1:1 ratio during the same study period. In the T > 3cm group, we included patients who underwent IR as cases. Patients who underwent TM were selected as controls, matching for age, pT, and pN in a 1:1 ratio.

We collected patients’ demographic data, clinicopathological data, and immunohistochemistry (IHC) results regarding estrogen receptor (ER), progesterone receptor (PR), human epidermal growth factor receptor 2 (Her2), P53, Ki67, and prognosis information.

The analytical results of breast cancer subtypes have been described elsewhere [17]. Subtypes in this study included luminal A (ER-positive [+] and/or PR+ and Her2− and Ki67 ≤14%), luminal B (ER+ and/or PR+ and Her2− and Ki67 > 14%; ER+ and/or PR+ and Her2+ and Ki67 anyway), Her2 overexpression (ER-negative [−], PR−, and Her2+), and triple-negative breast carcinoma (TNBC [ER−, PR−, and Her2−]). However, cases of Her2 with IHC (2+) and FISH (+) were excluded from the TNBC group. IHC was scored by two independent pathologists who were blinded to patient clinicopathological characteristics and outcomes. To eliminate nonspecific staining, a negative control was performed using phate-buffered saline (PBS).

All cases were evaluated by key stakeholders from a multidisciplinary consultation board with full adherence to updated international guidelines [18]. The follow-up period for each patient was estimated from the date of diagnosis of cancer to January, 2020 (the end of the study). Patients in each cohort followed the same clinical follow-up protocols, which were scheduled every 6 months.

### Outcomes

The primary endpoint was 5-year disease-free survival (DFS), which was defined from the date of surgery until relapse or the date patients were last known to be alive. The secondary outcome was patient satisfaction and the quality of life, which were evaluated using The Swedish Short Form-36 Health Survey (SF-36) [19]. SF-36 has been frequently used for health-related quality of life (HRQoL) assessments for patients with breast cancer [20].

### Statistical analysis

We constructed Kaplan-Meier curves to estimate the 5-year DFS. The log-rank test was used to assess differences between groups. *χ*^2^ test was used to compare clinicopathological characteristics between two groups. Statistical analyses were performed using SPSS version 17.0 (IBM Corporation, Armonk, NY, USA) for Windows (Microsoft Corporation, Redmond, WA, USA). A *P* value less than 0.05 was considered statistically significant.

## Results

**Fig. 2:**
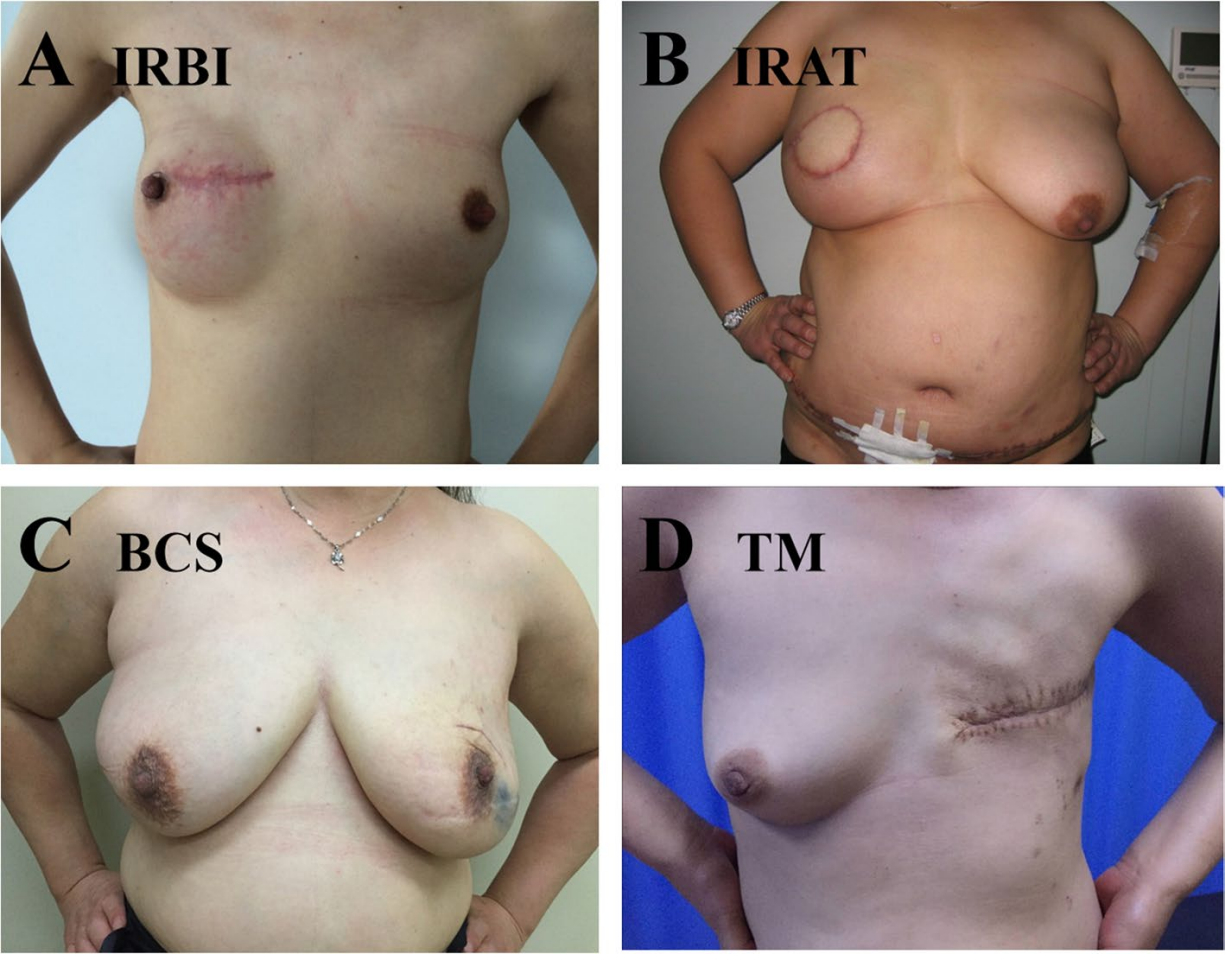
Post-operative positive photos. **A** Immediate reconstruction of breast implants (IRBI); **B** Immediate reconstruction of autologous tissue (IRAT); **C** Breast conserving surgery (BCS); **D** Total mastectomy (TM)

**Fig. 3:**
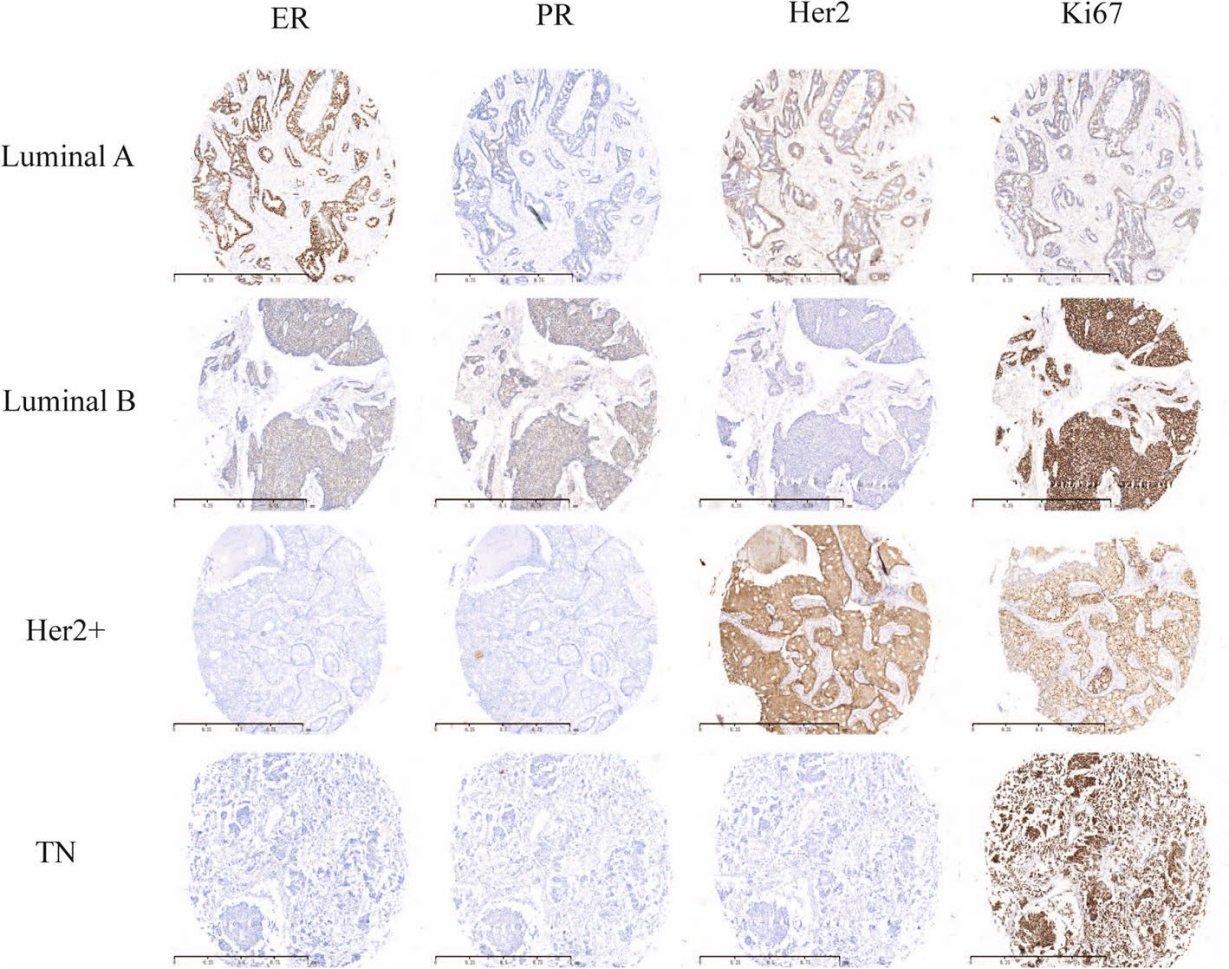
Expression of ER, PR, Her2, and Ki-67 by immunohistochemical staining in luminal A, luminal B, Her2+, and TN breast cancer (the same patient with the same lesion site of each type). Positive expression of ER, PR, and Ki67 revealed nuclear staining, original magnification×100. Positive expression of Her2 revealed membrane staining, original magnification of ×100

A total of 12,678 consecutive breast cancer patients who underwent various surgical procedures were extracted from the HMUCH database for the eligibility assessment, of which 216 cases underwent IR (155 cases in the T ≤ 3 cm group vs 61 cases in the T > 3cm group) meeting the study criteria were included in the final analysis. 155 cases of BCS and 155 of TM were matched with 155 cases of IR in the T ≤ 3 cm group, of which 118 performed breast implant (IRBI) (Fig. 2A) and 37 performed autologous tissue (IRAT) (Fig. 2B). The study groups were similar at baseline. 155 patients underwent IR were included in the final analysis with a median age of 37.24 years old (range 21–55) and 70 (45.16%) of them aged under 35 (Table 1). 56 patients were diagnosed with stage I breast cancer and 99 were at stage II. Analytical results of pathology features showed the median tumor size was 2.0 cm (range, 0.4–3.0 cm). T1 (75 [48.39%]) and T2 (80 [51.61%]) were the most common size of the study group. 110 (70.97%) patients had PN0, and 45 (29.03%) patients had N1-2. More than half (104 [67.10%]) of the patients in the study group had histological grade (HG) II at diagnosis. IHC staining results for protein expression in the four subtypes (luminal A, luminal B, Her2 overexpression, and TNBC) of breast cancer tissues were illustrated in Fig. 3. Luminal B (67 [43.23%]) was the most frequent subtype, followed by luminal A (55 [35.48%]), TN (17 [10.97%]), and Her2+ (16 [10.32%]). Of the 155 study participants, 105 cases received cytotoxic chemotherapy, 122 received hormone therapy (HT), and 19 underwent radiotherapy (RT). All these patients had at least four positive axillary lymph nodes.

**Table 1:** Patients’ clinicopathological features of IR, BCS, and TM in T≤3cm

Characteristic feature	IR (*N*=155)	BCS (*N*=155)	TM (*N*=155)	*P*
	*n* (%)	*n* (%)	*n* (%)	
Age				Matching variable
≤35	70 (45.16)	70 (45.16)	70 (45.16)	
>35	85 (54.84)	85 (54.84)	85 (54.84)	
pT				Matching variable
T1(≤2cm)	75 (48.39)	75 (48.39)	75 (48.39)	
T2(2–3cm)	80 (51.61)	80 (51.61)	80 (51.61)	
pN				Matching variable
N0	110 (70.97)	110 (70.97)	110 (70.97)	
N1-2	45 (29.03)	45 (29.03)	45 (29.03)	
Stage				
I	56 (36.13)	51 (32.91)	45 (29.03)	0.210
IIA	74 (47.74)	77 (49.68)	70 (45.16)	
IIB	25 (16.13)	27 (17.41)	40 (25.81)	
HG				0.906
I	38 (24.52)	42 (27.10)	41 (26.45)	
II	104 (67.10)	101 (65.16)	98 (63.23)	
III	13 (8.39)	12 (7.74)	16 (10.32)	
ER				0.186
ER (+)	113 (72.90)	101 (65.16)	99 (63.87)	
ER (−)	42 (27.10)	54 (34.84)	56 (36.13)	
PR				0.520
PR (+)	106 (68.39)	99 (63.87)	108 (69.68)	
PR (−)	49 (31.61)	56 (36.13)	47 (30.32)	
Her2				0.266
Her2 (+)	36 (23.23)	46 (29.68)	48 (30.97)	
Her2 (−)	119 (76.77)	109 (70.32)	107 (69.03)	
Ki67				0.318
Ki67 (≤14%)	76 (49.03)	67 (43.23)	80 (51.61)	
Ki67 (>14%)	79 (50.97)	88 (56.77)	75 (48.39)	
Subtype				0.990
Luminal A	55 (35.48)	57 (36.77)	51 (32.90)	
Luminal B	67 (43.23)	62 (40.00)	67 (43.23)	
Her2+	16 (10.32)	18 (11.61)	18 (11.61)	
TN	17 (10.97)	18 (11.61)	19 (12.26)	
Chemotherapy				0.686
Yes	102 (65.81)	100 (64.52)	107 (69.03)	
No	53 (34.19)	55 (35.48)	48 (30.97)	
Hormone therapy				0.917
Yes	122 (78.71)	120 (77.42)	119 (77.77)	
No	33 (21.29)	35 (22.58)	36 (23.23)	
Anti-Her2 therapy				0.940
Yes	31 (86.11)	39 (84.78)	40 (83.33)	
No	5 (13.89)	7 (15.22)	8 (16.67)	
Local recurrence				0.919
Yes	12 (7.74)	13 (8.38)	14 (9.03)	
No	143 (92.26)	142 (91.62)	90.97	
Distant metastasis				0.531
Yes	10 (6.45)	11 (7.09)	15 (9.67)	
No	145 (93.55)	144 (92.91)	140 (90.33)	

*Abbreviations*: *pT* pathological tumor size, *pN* pathological nodal status, *HG* histological grade, *ER* estrogen receptor, *PR* progesterone receptor, *TN* triple negative, *IR* immediate reconstruction, *BCS* breast conservation surgery, *TM* total mastectomy

Sixty one cases of TM were matched with 61 cases of IR in the T > 3cm group (Fig. 1 and Fig. 2A–D). Analyses of clinicopathological features were summarized in Table 2. 33 (54.10%) women aged under 35 years old. 21 patients were diagnosed with stage II breast cancer and 40 were at stage III. The median tumor size was 4.0 cm (range, 3.1–5.6 cm). 45 (74.77%) patients had PN1-2 and 16 (26.23%) had N0. More than half of the patients in the study group had HG II at diagnosis (77.05% [*n* = 47]). Luminal B (24 [39.34%]) was the most frequent subtype, followed by TN (14 [22.95%]), Her2+ (13 [21.31%]), and luminal A (10 [16.39%]). Most patients underwent chemotherapy with HT and RT.

**Table 2:** Patients’ clinicopathological features of IR and TM in T>3cm

Characteristic feature	IR (*N*=61)	TM (*N*=61)	*P*
	*n* (%)	*n* (%)	
Age			Matching variable
≤35	33 (54.10)	33 (54.10)	
>35	28 (45.90)	28 (45.90)	
pT			Matching variable
T2(>3cm, ≤5cm)	46 (75.41)	46 (75.41)	
T3(>5cm)	15 (24.59)	15 (24.59)	
pN			Matching variable
N0	16 (26.23)	16 (26.23)	
N1-3	45 (73.77)	45 (73.77)	
Stage			0.706
II	21 (34.43)	23 (37.70)	
III	40 (65.57)	38 (62.30)	
HG			0.820
I	6 (9.84)	7 (11.48)	
II	47 (77.05)	44 (72.13)	
III	8 (13.11)	10 (16.39)	
ER			0.691
ER (+)	44 (72.13)	42 (68.85)	
ER (-)	17 (27.87)	19 (31.15)	
PR			0.455
PR (+)	36 (59.02)	40 (65.57)	
PR (-)	25 (48.98)	21 (34.43)	
Her2			0.700
Her2 (+)	21 (34.43)	19 (31.15)	
Her2 (-)	40 (65.57)	42 (69.85)	
Ki67			0.817
Ki67 (≤ 14%)	11 (16.39)	12 (19.67)	
Ki67 (>14%)	50 (83.61)	49 (80.33)	
Subtype			0.986
Luminal A	10 (16.39)	9 (36.77)	
Luminal B	24 (39.34)	25 (40.98)	
Her2+	13 (21.31)	14 (22.95)	
TN	14 (22.95)	13 (21.31)	
Chemotherapy			0.638
Yes	49 (65.81)	51 (83.61)	
No	12 (34.19)	10 (16.31)	
Hormone therapy			0.691
Yes	42 (68.85)	44 (72.13)	
No	19 (31.15)	17 (27.87)	
Radiotherapy			0.277
Yes	29 (47.54)	35 (57.38)	
No	32 (52.46)	26 (42.62)	
Anti-Her2 therapy			0.894
Yes	18 (85.71)	16 (84.21)	
No	3 (14.29)	3 (15.79)	
Local recurrence			0.126
Yes	12 (19.67)	6 (9.83)	
No	49 (80.33)	55 (90.16)	
Distant metastasis			0.408
Yes	9 (14.75)	6 (9.83)	
No	52 (85.25)	55 (90.17)	

*Abbreviations*: *pT* pathological tumor size, *pN* pathological nodal status, *HG* histological grade, *ER* estrogen receptor *PR* progesterone receptor, *TN* triple negative, *IR* immediate reconstruction, *BCS* breast conservation surgery, *TM* total mastectomy

**Fig. 4:**
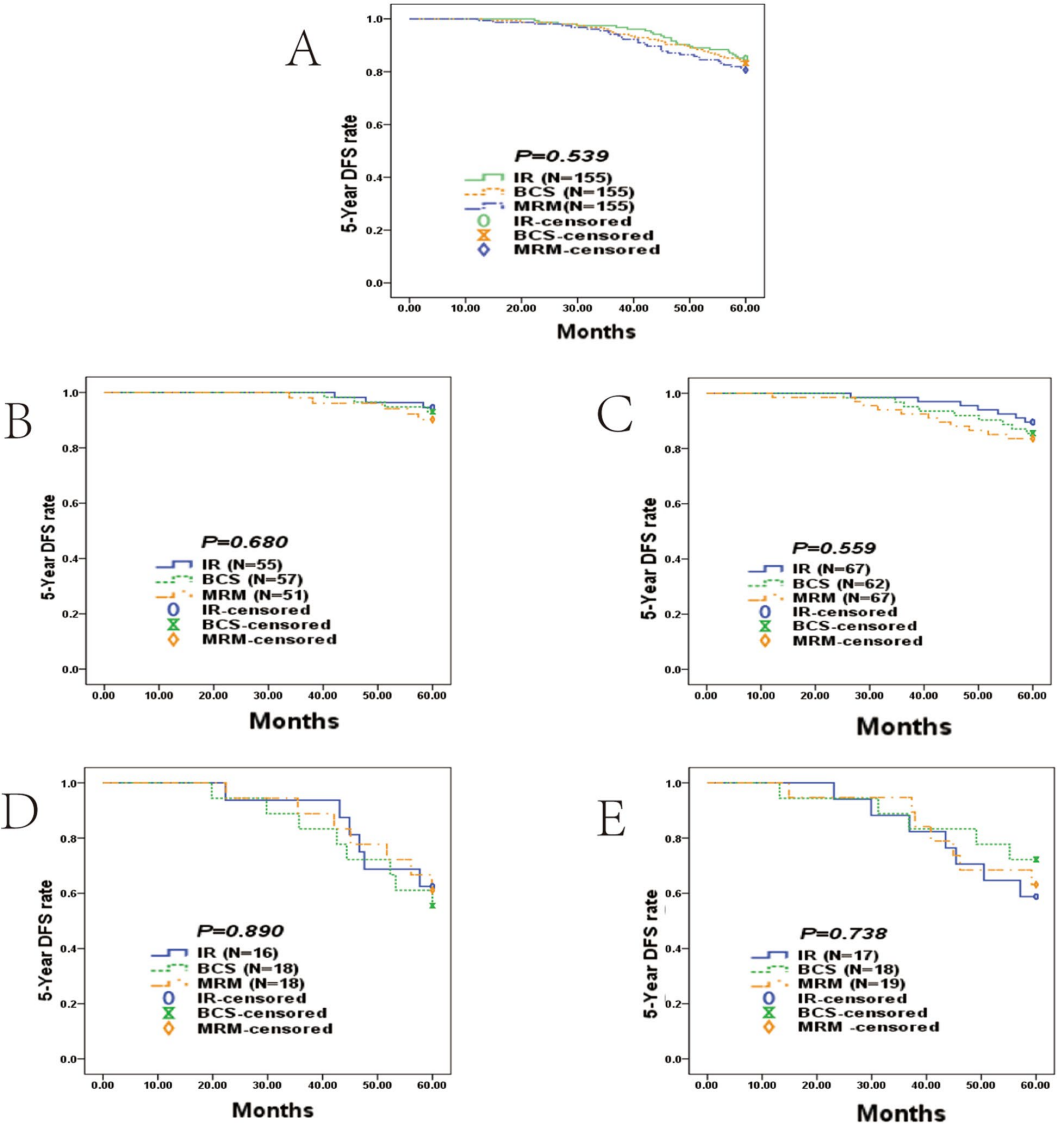
Survival curves in the T≤3cm group. **A** Kaplan-Meier analysis for 5-year DFS curves based on IR (*N*=155), BCS (*N*=155), and TM (*N*=155) in the T≤3cm group; **B** 5-year DFS curves stratified by four subtypes in luminal A; **C** 5-year DFS curves stratified by four subtypes in Luminal B; **D** 5-year DFS curves stratified by four subtypes in Her2+; and **e** 5-year DFS curves stratified by four subtypes in TN

No statistical differences were observed in terms of 5-year DFS for the three different surgical techniques performed in patients with T≤3 cm lesions (Table 3, Fig. 4A). In addition, patients of luminal A, luminal B, Her2 overexpression, and TNBC subtype had similar 5-year DFS rates (Fig. 4B–E).

**Table 3:** Kaplan–Meier analysis for 5-year DFS based on 4 different subtypes on IR, BCS, and TM (log-rank test) in T≤3cm group

	5-Y DFS	Median 5-Y DFS		*P*
	%	Months	95%CI	
Total				0.539
IR	85.2%	57.73	56.65-58.80	
BCS	83.2%	57.16	55.87-58.46	
TM	80.6%	56.44	55.02-57.86	
Luminal A				0.680
IR	94.5%	59.42	58.66-60.18	
BCS	93.0%	59.23	58.36-60.10	
TM	90.2%	58.74	57.41-60.07	
Luminal B				0.559
IR	89.6%	58.67	57.44-59.91	
BCS	85.5%	57.75	56.05-59.45	
TM	83.6%	56.40	54.14-58.67	
Her2 +				0.890
IR	62.5%	53.89	48.88-58.91	
BCS	55.6%	52.11	46.12-58.09	
TM	61.1%	54.04	49.17-58.92	
TN				0.738
IR	58.8%	52.14	46.57-57.71	
BCS	72.2%	53.64	47.70-59.57	
TM	63.2%	52.69	47.22-58.16	

*IR* immediate reconstruction, *BCS* breast conservation surgery, *TM* total mastectomy, *TN* triple negative

**Fig. 5:**
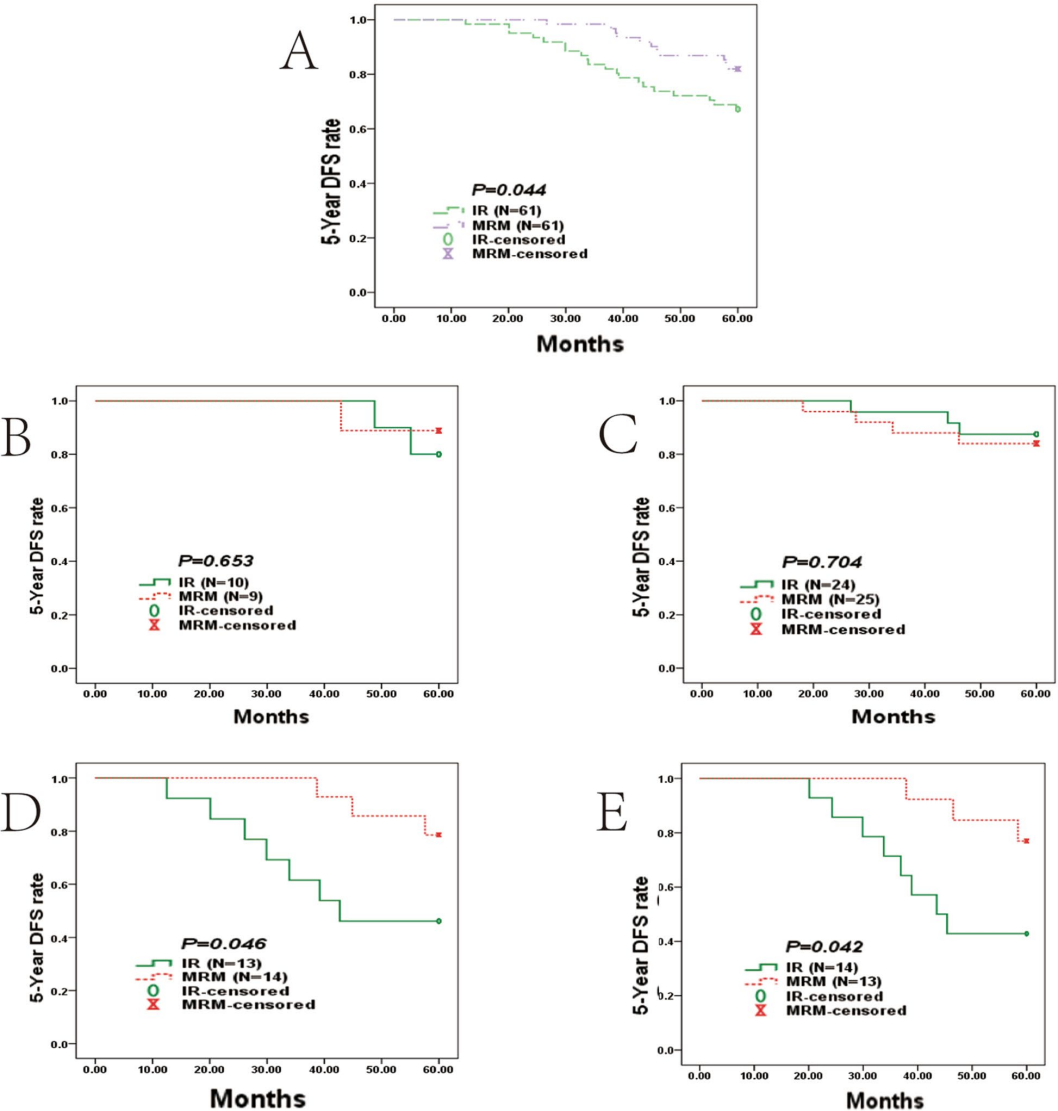
Survival curves in the T>3cm group. **A** Kaplan-Meier analysis for 5-year DFS curves based on IR (*N*=61) and TM (*N*=61) in the T>3cm group; **B** 5-year DFS curves stratified by four subtypes in luminal A; **C** 5-year DFS curves stratified by four subtypes in luminal B; **D** 5-year DFS curves stratified by four subtypes in Her2+; **E** and 5-year DFS curves stratified by four subtypes in TN

However, patients with T>3cm who received IR had an overall worse median 5-year DFS than those who received TM (52.28 months, 95% CI [48.99–55.58] vs 57.32 months, 95% CI [55.59–56.26]; *P* < 0.044; Fig. 5A and Table 4). The difference in the median 5-year DFS in the Her2+ cohort was marginally significant (57.23 months 95% CI [53.86–60.60] in the TM group vs 43.41 months 95% CI [34.19–52.64] in the IR group; *P* = 0.046; Fig. 5D). Patients in the TNBC cohort had a longer median 5-year DFS (57.14 months 95% CI [53.55–60.73] in the TM group vs 45.20 months 95% CI [37.72–52.68] in the IR group; *P* = 0.042; Fig. 5E).

**Table 4:** Kaplan–Meier analysis for 5-year DFS based on 4 different subtypes on IR, BCS, and TM (log-rank test) in T>3cm group

	5-Y DFS	Median 5-Y DFS		*P*
	%	Months	95%CI	
Total				0.044
IR	67.2%	52.28	48.99-55.58	
TM	82.0%	57.32	55.59-56.26	
Luminal A				0.653
IR	80.0%	58.39	56.21-60.57	
TM	88.9%	58.10	54.59-61.61	
Luminal B				0.704
IR	87.5%	57.37	54.33-60.41	
TM	84.0%	55.44	51.04-59.84	
Her2 +				0.046
IR	46.2%	43.41	34.19-52.64	
TM	78.6%	57.23	53.86-60.60	
TN				0.042
IR	42.9%	45.20	37.72-52.68	
TM	76.9%	57.14	53.55-60.73	

*IR* immediate reconstruction, *BCS* breast conservation surgery, *TM* total mastectomy, *TN* triple negative

### Aesthetic outcomes

Of the 155 eligible patients, the overall response rates were 82.58% (128), 83.87% (130), and 77.42% (120), respectively, for patients in the IR, BCS, and TM group (Fig. 1 and Table 5). Patients underwent IR were more satisfied than those who underwent BCS or TM in terms of skin quality/color (*P* < 0.001). Satisfaction scores regarding symmetry (*P* < 0.001), breast contour/size/position (*P* < 0.001), and nipple contour/size/position (*P* < 0.001) were also different among the three comparing groups. In addition, statistical differences were observed in terms of psychosocial well-being (*P* < 0.001) and sexual well-being (*P* < 0.001) among patients who underwent IR, BCS, and TM (Table 5). IR and BCS preserved improved aesthetic satisfaction, psychosocial well-being, and sexual well-being in patients with lesions size of ≤3 cm. However, there was no significant difference in complications (including necrosis, infection, hematoma/seroma, and prolonged wound healing) and impact on the detection of local recurrence among the three groups.

**Table 5:** Aesthetic and complications related quality-of-life outcomes according to IR, BCS, and TM in T≤3cm

	IR (*N*=128)	BCS (*N*=130)	TM (*N*=120)	*P*
	*n* (%)	*n* (%)	*n* (%)	
Aesthetic satisfaction				
Skin quality/color				<0.001
Satisfactory	106 (82.81)	99 (76.15)	69 (57.51)	
Medium	14 (10.94)	19 (14.62)	22 (18.33)	
Unsatisfactory	8 (6.25)	12 (9.23)	29 (24.16)	
Symmetry				
Satisfactory	118 (92.19)	122 (93.84)	2 (1.67)	<0.001
Medium	6 (4.69)	5 (3.85)	8 (6.67)	
Unsatisfactory	4 (3.12)	3 (2.31)	110 (91.66)	
Breast contour/size/position				<0.001
Satisfactory	116 (90.62)	120 (92.31)	1 (0.83)	
Medium	7 (5.47)	6 (4.61)	7 (5.84)	
Unsatisfactory	5 (3.91)	4 (3.08)	112 (93.33)	
Nipples contour/size/position				<0.001
Satisfactory	101 (78.91)	110 (84.62)	1 (0.83)	
Medium	12 (9.37)	11 (8.46)	5 (4.17)	
Unsatisfactory	15 (11.72)	9 (6.92)	114 (95.00)	
Complications				
Necrosis				0.621
Yes	4 (3.22)	2 (1.54)	4 (3.33)	
No	124 (96.88)	128 (98.46)	116 (96.67)	
Infection				0.761
Yes	6 (4.68)	5 (3.85)	7 (5.83)	
No	122 (95.32)	125 (96.15)	113 (94.17)	
Hematoma/seroma				0.981
Yes	11 (8.59)	12 (9.23)	11 (9.17)	
No	117 (91.41)	118 (90.77)	109 (90.83)	
Prolonged wound healing				0.459
Yes	4 (3.22)	2 (1.54)	5 (4.17)	
No	124 (96.88)	128 (98.46)	115 (95.83)	
Psychosocial well-being				<0.001
Good	91 (71.09)	106 (81.54)	15 (12.50)	
Medium	27 (21.09)	17 (13.08)	26 (21.67)	
Poor	10 (0.78)	7 (5.38)	79 (65.83)	
Sexual well-being				<0.001
Good	90 (70.31)	95 (73.07)	11 (9.17)	
Medium	26 (20.31)	25 (19.23)	21 (17.50)	
Poor	12 (9.38)	10 (7.69)	88 (73.33)	

## Discussion

This retrospective match-cohort study has provided information on long-term oncologic follow-up outcomes for 587 patients with invasive breast cancer at the Harbin Medical University Cancer Hospital in China.

Patients underwent IR can achieve satisfactory oncological and cosmetic outcomes even with a lesion > 2 cm tumor size [21]. IR allows all breast excisions and prevents breast deformities by wide resection defects; therefore, it improves patients’ cosmetic outcomes [22–24]. Based on our observation, improved aesthetic satisfaction, and psychosocial and sexual well-being were obtained in those who underwent IR and BCS in the T ≤3 cm group.

Reducing the incidence of complications is one of the goals of breast cancer procedures. Our study showed there was no significant difference in complications among IR, BCS, and TM (including necrosis, infection, hematoma/seroma, and prolonged wound healing). In recent years, many studies were trying to reduce the complications by improved procedures. Liu et al. showed that compared with traditional mastoscopic modified radical mastectomy (MRM), MRM with skin nipple-areola preservation under air cavity-free suspension hook and stage I silicone prosthesis implantation (SMALND) had lower complication rates [25]. For robotic surgery, dissection with non-robotic scissors and then robotic dissection can reduce the skin complication rate than dissection with robotic scissors [26]. For breast-conserving surgery with acellular dermal matrix (ADM), appropriate planning of skin incision that should be performed as far away from the tumor site as possible can minimize the risk of ADM extrusion [27, 28]. Immediate free flap reconstruction is a feasible and safe option with low risk of complications that result in a soft and natural-shaped breast [29]. Lagergren J [30] had 13% local complications in a 5-year follow-up among 249 women with cancer operated with immediate breast reconstruction. Complication rates are acceptably low with levels similar to other published materials [4, 5, 8]. We observed a low initial complication rate but, compared with contant C [31], our complications seem higher if we include the cosmetic corrections. Surgical procedures of IR, which include more excision of the skin and glands, are typically complicated for patients with breast cancer lesions > 3 cm. This will subsequently increase the risk of complications associated with additional adjuvant therapies and local recurrence. Takaaki [32] recommend that in cases with invasive breast cancer in which the dermis-to-tumor distance is less than 2 mm, the skin immediately overlying the tumor be resected in patients who underwent IBR with skin-sparing (SSM) or nipple-sparing mastectomy (NSM).

Survival rate and recurrence rate are important indicators of oncological safety. As adjuvant therapies continue to improve and long-term survival rates increase, patient preferences and quality of life indicators have become increasingly important determinants for treatment options [33]. Several studies report no significant differences in recurrence patterns or incidences between patients undergoing a skin-sparing mastectomy followed by IR with those of conventional mastectomy [34, 35]. In Lee SB study, the IR group showed better breast cancer-specific survival rate and distant metastasis-free survival rate, outcomes than the conventional mastectomy group [36]. This may suggest selection bias resulting from the patients in this group wanting to avoid factors, which can have a negative influence on 5-year DFS. Factors influencing 5-year DFS were tumor size, node metastasis, stage, lymphovascular invasion, type of surgery, chemotherapy, and radiation therapy, according to univariate analysis.

Vahit [37] show that the patients who underwent partial mastectomy (PM) with mini latissimus dorsi flap (MLDF) reconstruction had a significantly superior cosmetic outcome and a better regarding quality of life as compared to the patients who had a subcutaneous mastectomy with implant (M + I) reconstruction. Despite the advances and developments in BCS, a recent trend towards patient’s preference for TM or IR has emerged in some countries [38]. Our study highlights that patients underwent IR at a relatively young age, meaning IR is particularly applicable in young patients who are urgently willing to have their breast reconstructed after mastectomy. In Asian breast cancer patients, the proportion of patients in younger than 35 age group was reported to be much higher (ranging from 9.5 to 12%) [39]. However, it accounts for fewer than 4% of the total number of breast cancer cases diagnosed in Western countries [40, 41]. In our study, the percentage of young patients under 35 years old was 45.16%. In general, younger patients are more concerned about their body image than older patients. An important factor affecting survival is the difference in the proportion of patients who tend to be younger.

Our study showed that IR technique is a safe and reliable surgical treatment for managing T≤3 cm invasive breast cancers lesion. However, since tumor size > 3 cm is associated with an increase of local recurrence, especially in those with TNBC and Her2+ subtypes, IR surgical technique applies to breast cancer patients with T > 3 cm lesions should be with great caution. Several meta-analyses have concluded that IR is an oncologically safe procedure, at least for patients with early-stage disease and a small tumor [42, 43]. However, different results have also been obtained for patients with tumor size > 3 cm. Despite the relatively small number of patients with T > 3 cm lesions in this study, our findings strengthen the opinion that surgical technique followed by IR may be cautious for patients with a locally advanced-stage disease with TNBC and Her2+ subtypes.

One of the major strengths of our study is our study was the first study to examine the survival rate of four different subtypes (luminal A, luminal B, TNBC, and Her2 overexpression) of breast cancer patients who underwent IR. These subgroup analyses enabled us to precisely identify the population that is most suitable for IR.

To the best of our knowledge, there was only one study to date using a pair-matched study design to compare TM with BCS in patients with tumor size > 2 cm [44]. However, no long-term oncological follow-up and comparison with patients undergoing IR have been published. Our study was the first to directly compare IR with BCS and TM in consecutive IDC patients who underwent surgery during similar time periods.

Our study had several limitations. First, the single-center study design and a relatively small sample size limited the generalization of the conclusions; therefore, more data are needed to confirm the results. However, despite no statistical difference, we did find that patients who underwent IR had a marginally longer 5-year DFS compared with TM and BCS in the T≤3cm group, which is probably due to the small sample size either. Potential clinical significance might be observed in future long-term practice. Second, insufficient cases limited our analysis of the difference between recurrence and metastasis in different surgical methods. Third, we only matched age, pathological tumor size, and pathologic nodal status for controlling confounders. However, there might be other potential confounders that we did not consider. Finally, the study population was restricted to females, and therefore, our findings cannot be directly generalizable to male patients.

## Conclusion

Our findings indicate that for patients with T≤3 cm invasive breast cancer, IR had no statistical difference compared with BCS or TM in terms of DFS. However, it can improve patients’ aesthetic outcomes, psychosocial, and sexual well-being. For patients with T > 3 cm invasive breast cancer, TM is superior to IR as it predicts a better 5-year DFS.

## Data Availability

The raw data of this manuscript are available upon reasonable request from the corresponding author.

## Abbreviations

IR: Immediate reconstruction
BCS: Breast conservation surgery
TM: Total mastectomy
pT: Pathological tumor size
TN: Triple negative
pN: Pathological nodal status
HG: Histological grade
ER: Estrogen receptor
PR: Progesterone receptor
