# Correction to: Long-term oncologic safety of immediate reconstructive surgery in patients with invasive breast cancer: a retrospective matched-cohort study

**DOI:** 10.1186/s12957-022-02523-3

**Published:** 2022-02-28

**Authors:** Yanni Song, Shanshan Sun, Dalin Li, Jiguang Han, Ming Niu, Sai Luo, Haiqian Xu, Rui Huang, Sihang Zhang, Yang Wu, Qiqi Wu, Jing Xiong, Lijun Hao

**Affiliations:** 1 Department of Plastic and Cosmetic Surgery, The First Affiliated Hospital of Harbin Medical University, 141 Yiman Road, Harbin, 150010 China; 2 Department of Breast Surgery, Harbin Medical University Cancer Hospital, 150 Haping Road, Harbin, 150081 China; 3 Department of Pathology, Tianjin Medical University Cancer Hospital, 1 Huanhu Xi Road, Tianjin, 300060 China; 4 Department of General Surgery, The Second Affiliated Hospital of Harbin Medical University, 246 Xuefu Road, Harbin, 150086 China; 5 Department of Epidemiology and Bio-Statistics, School of Public Health, Peking University Health Science Center, 38 Xueyuan Road, Haidian District, Beijing, China; 6 Sino-Russian Medical Research Center, Heilongjiang Academy of Medical Sciences, 157 Baojian Road, Harbin, China; 7 Northern (China) Translational Medicine Research and Cooperation Center, Heilongjiang Academy of Medical Sciences, 157 Baojian Road, Harbin, China

**Correction to: World Journal of Surgical Oncology 19, 348, (2021)**

**https://doi.org/10.1186/s12957-021-02450-9**

Following the publication of the original article [1], the author reported that the images were not renumbered to conform with the Figure citations and legends. The image for Fig. 1 should be the one in Fig. 3. The image in Fig. 2 should be the one in Fig. 1 and the image in Fig. 3 should be the one in Fig. 2. The correct figures are included here.

**Fig. 1:**
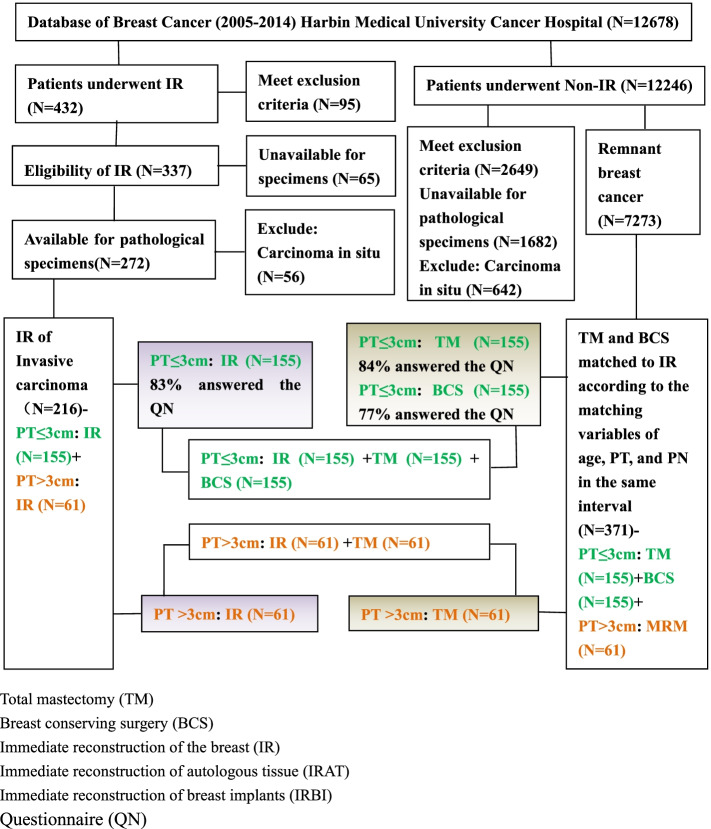
Study object. Patients were selected from the database of the Breast Cancer Center between 2005 and 2014. IR immediate reconstruction, IRBI immediate reconstruction of breast implants, IRAT immediate reconstruction of autologous tissue, BCS breast-conserving surgery, TM total mastectomy

**Fig. 2:**
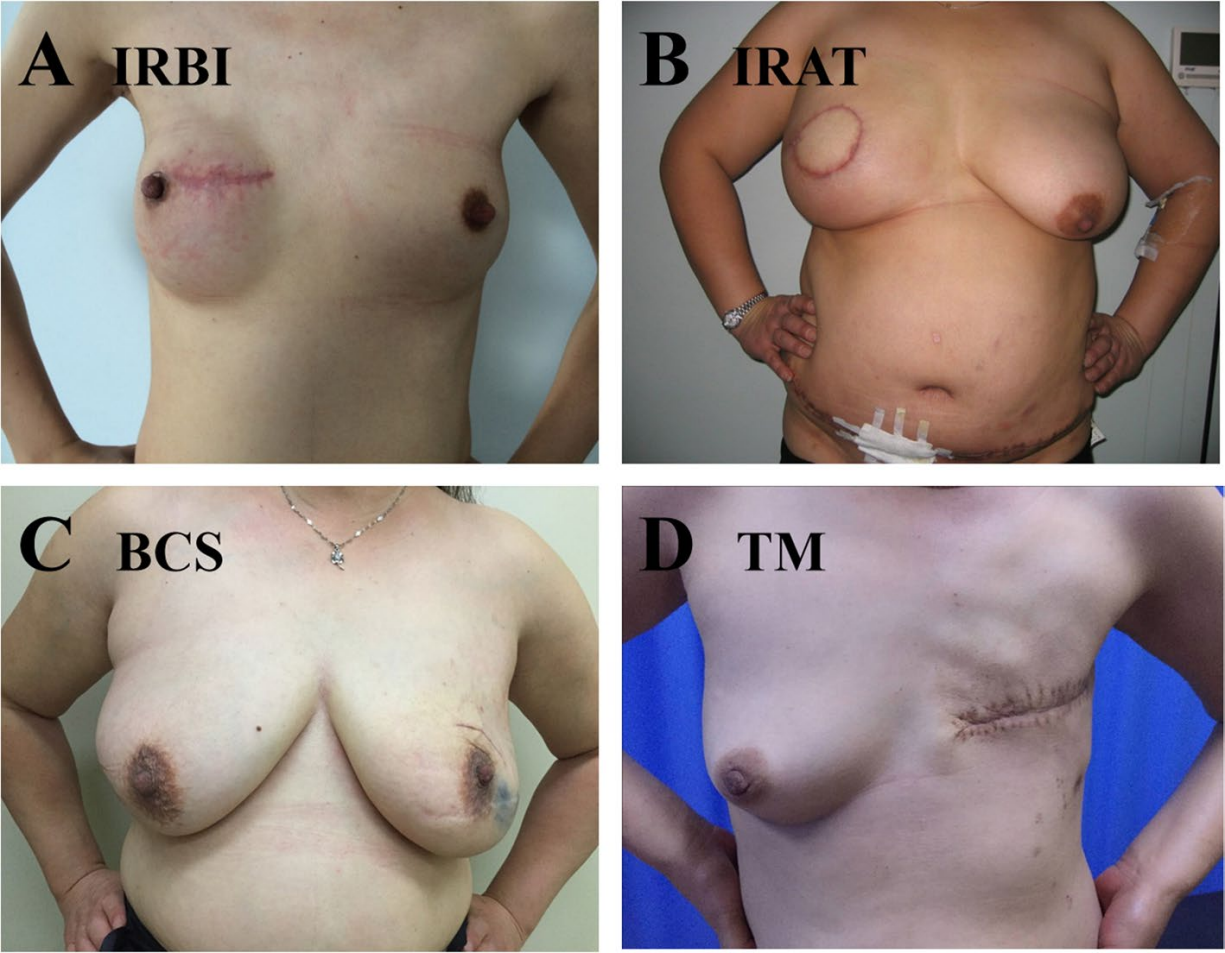
Post-operative positive photos. **A** Immediate reconstruction of breast implants (IRBI); **B** Immediate reconstruction of autologous tissue (IRAT); **C** Breast conserving surgery (BCS); **D** Total mastectomy (TM)

**Fig. 3:**
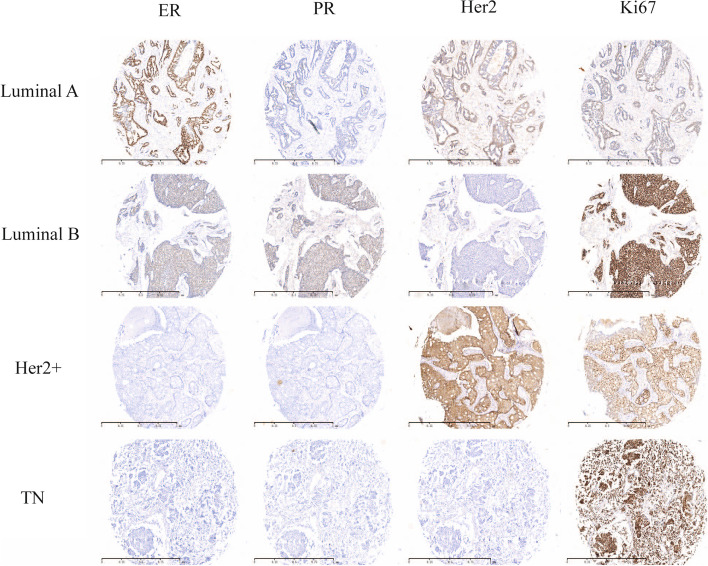
Expression of ER, PR, Her2, and Ki-67 by immunohistochemical staining in luminal A, luminal B, Her2+, and TN breast cancer (the same patient with the same lesion site of each type). Positive expression of ER, PR, and Ki67 revealed nuclear staining, original magnification×100. Positive expression of Her2 revealed membrane staining, original magnification of ×100

The original article has been updated.
